# Comparing the Effects of Virtual Reality Breathing Exercise and Incentive Spirometry Exercise on Improving Pulmonary Function in Children with Spastic Diplegic Cerebral Palsy

**DOI:** 10.7759/cureus.59149

**Published:** 2024-04-27

**Authors:** Rajesh S, Vadivelan Kanniappan, B.S. Santhosh Kanna, Veeragoudhaman T. S.

**Affiliations:** 1 Physiotherapy, SRM (Sri Ramaswamy Memorial) College of Physiotherapy, SRM Institute of Science and Technology, Chengalpattu, IND; 2 Physiotherapy, National Institute for Empowerment of Persons with Multiple Disabilities (Divyangjan) - Government of India, Chennai, IND

**Keywords:** cerebral palsy, pulmonary function test, incentive spirometer, virtual reality breathing training, spastic diplegic

## Abstract

Introduction: Children with cerebral palsy (CP) have a higher incidence of respiratory dysfunction than healthy children. Virtual reality breathing therapy is an assistive technology that is becoming popular in the rehabilitation of children with CP.

Methods: This experimental study included a total of 32 children with spastic diplegic CP who were divided into two groups: the virtual reality breathing training (VRBT) group and the incentive spirometry (IST) group. Individuals classified as levels I to III on the gross motor function classification system (GMFCS) were recruited using the simple random sampling method.

Result: The results of comparing the values of forced vital capacity (FVC), forced expiratory volume at one second (FEV1), and the ratio of FVC/FEV1 showed a significant difference between groups. A significant difference was found in the VRBT group compared to the IST group, except for the peak expiratory flow (PEF) values, which showed a nonsignificant difference between the groups.

Conclusion: There were significant differences in FVC and FEV1 between the VRBT and IST groups. It has been concluded that VRBT has additional benefits in improving pulmonary functions.

## Introduction

Children with cerebral palsy (CP) have a higher incidence of respiratory dysfunction than healthy children [1]. Children with spastic CP have around 40% of impairment in the respiratory function [2]. CP does not directly cause dysfunction of lung parenchyma or airways; however, pulmonary function is reduced in children with CP due to the involvement of the nervous system, which also affects the lungs and cardiopulmonary status [3]. Atelectasis, sleep apnea, pneumonia, and chronic obstructive pulmonary disease (COPD) are the associated problems in children with CP [4]. Therefore, the mortality and morbidity rates of children with CP can be reduced by improving pulmonary function.

Impaired mobility of the chest wall, respiratory muscle weakness, and alteration in the chest wall alignment reduce the pulmonary function of children with spastic CP [5]. Virtual reality (VR) therapy is a recently popular assistive technology in the rehabilitation of children with CP [6]. VR is primarily used to improve structural and functional body involvement such as range of motion, hand function, balance and gait training, and patients’ confidence and motivation. In a study by Joo et al., VR breathing training (VRBT) was used for stroke patients, which reduced breathlessness and improved exercise performance and quality of life of the patients [7]. Another study states that VRBT improves the strength of respiratory muscle for patients with non-cystic fibrosis bronchiectasis [8]. Patients with neurological impairment to motor involvement show better outcomes with treatments such as biofeedback [7]. The main advantage of VRBT is that it creates normal movement patterns in neurological conditions like that in stroke patients [7]. Audio and visual images were used earlier in biofeedback. At present, the treatment techniques take into account the patient’s interests and daily living activities, which is more helpful with VR treatment [9-11]. VRBT improves the strength of muscles of respiration, which is the alternative for respiratory muscle training in bronchiectasis patients [8].

Several studies have reported that VR therapy can provide maximal efficacy in improving motor function and quality of life. However, few studies have considered VR therapy as a treatment approach for improving the pulmonary function of children with CP. The aim of this study is to find out the effectiveness of VRBT in improving the pulmonary function of children with spastic diplegic CP.

## Materials and methods

This experimental study was conducted at the Department of Therapeutics, Physiotherapy Division, National Institute for Empowerment of Persons with Multiple Disabilities (Divyangjan), Governemnt of India, Chennai, Tamil Nadu, India. The study was approved by the Ethical Committee of the National Institute for Empowerment of Persons with Multiple Disabilities (approval number: NIEPMD/R&D.20(4)/2019). Written informed consent was taken from the parents or caregivers of children with CP.

A total of 32 children with a diagnosis of spastic diplegic CP in the age group of 6-15 years and a Gross Motor Function Classification Scale (GMFCS) level of I to III were included in this study. Patients were excluded if they underwent surgery for orthopedic complications or were administered botulinum toxin within the past six months. Those with acute or chronic pulmonary disease, recent medical illnesses, reduced walking ability due to musculoskeletal problems, or a reduced intelligence quotient assessed using the Stanford Binet Intelligence scale by a clinical psychologist, which interfered with study participation, were excluded. The patients were divided into two groups. Group I received VRBT through Breathing Trainer, and Group II received incentive spirometry training (IST).

VRBT procedure

**Figure 1:**
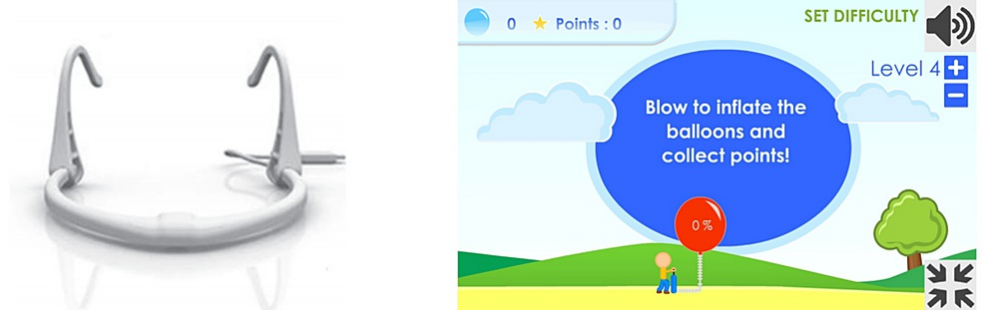
Device* used for the virtual reality breathing training group and the training screen. *Breathing+ package (Breathing Labs, Ljubljana, Slovenia)

The Breathing+ package (Breathing Labs, Ljubljana, Slovenia), was used for this study. The software, which contains various games suitable for children, was downloaded (Figure 1). Before starting the game, proper instruction was given to patients for inhalation and exhalation. Rest periods of 30 seconds were given in between repetitions of the treatment.

**Figure 2:**
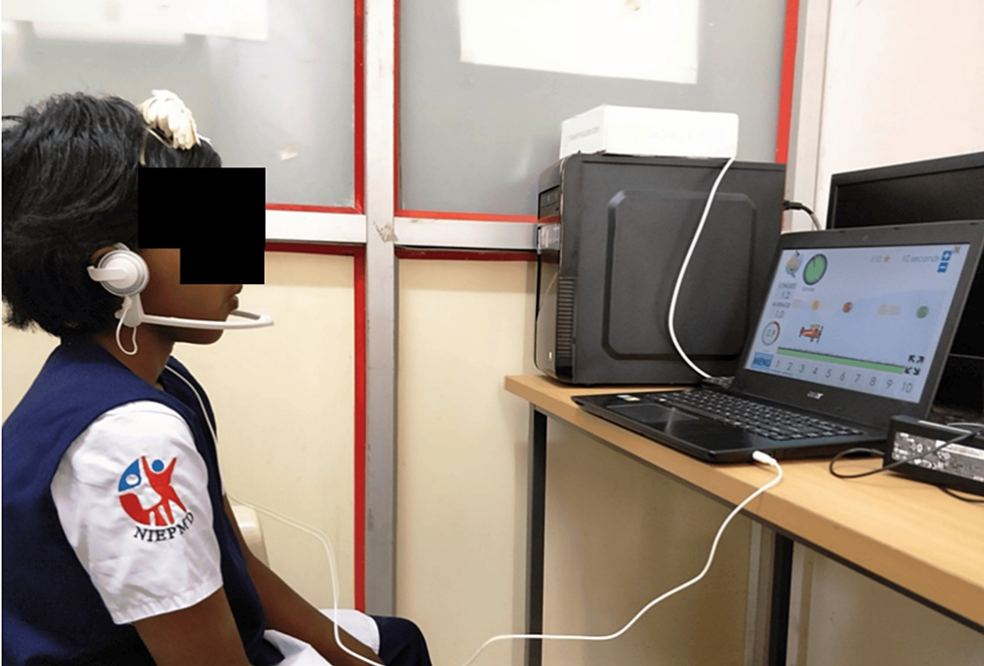
Virtual Reality Breathing Training

The application included 14 games, such as windmill blowing, kite flying, and balloon blowing. In each game, about 10 sets of inhalation and prolonged exhalation were included; at the end, the average of the total value was taken. The choice of the game was given to patients, along with the decision to use a desktop or mobile phone. The patient was seated in an armrest chair in a well-supported position to prevent falls or giddiness. Instructions were given by the therapist. Before beginning, the game was demonstrated, and the therapist monitored the patient until the end of the session. The patient was asked to perform a long exhalation to show good pulmonary function. The training screen seen by the patient offered good visual feedback (Figure 2). The duration of the treatment session was around 25 minutes, and five minutes of breathing control exercises were performed at the beginning and end of the session. Each set carried 10 repetitions and in between each repetition, 30 seconds rest period was given. The VRBT group received treatment five days a week for six weeks for approximately 25 minutes per day.

IST procedure

The children in the IST group were provided with instructions on how to do the incentive spirometry exercise. The proper usage involved holding the incentive spirometer upright, sealing the lips tightly around the mouthpiece, and taking a slow and deep breath. The patient was encouraged to reach a specific volume through visual feedback. Holding the breath for two to three seconds at full inspiration was recommended, followed by slow expiration. The exercise was to be performed 10 times with three sets per session for five days a week, for a duration of six weeks [12].

Conventional exercise includes mat activities, active mobility exercises, strengthening exercises of the lower limb, and muscle elongation exercises for muscle tightness, along with treadmill training with zero inclination, static bicycle training, or parallel bar walking. All these exercises were performed five minutes in each session, five days a week [13]. Conventional exercises were done by both groups. At the beginning and end of the six weeks, all patients underwent pulmonary function tests (PFTs).

Tests and scales

Gross Motor Function Classification System

The scale measures the gross motor function of children with CP into five levels, which range from being able to walk independently to being wheelchair dependent [14].

PFT

A PFT was performed with a Helios-401 spirometer (Recorders & Medicare Systems Pvt. Ltd., Panchkula, Chandigarh, India) to measure pulmonary function. The following parameters were tested: forced vital capacity (FVC), forced expiratory volume at one second (FEV1), FVC/FEV1 ratio, and peak expiratory flow (PEF). Patients were instructed to take deep breaths and exhale through the device. To prevent air from escaping through the nasal passage, a soft nose clip was applied.

Statistical analysis

Statistical analysis was done using PASW Statistics for Windows, Version 18.0 (Released 2009; SPSS Inc., Chicago, United States). Paired and unpaired t-tests were done to determine the significance. Pre- and post-test effects were calculated to find out the differences between the means of the groups.

## Results

The pre- and post-test results showed significant improvement in pulmonary function within both groups, as shown in Table 1. This indicates that both interventions were effective in improving pulmonary function. A significant difference was found in the VRBT group’s values when comparing the values of FVC, FEV1, and FVC/FEV1 between the groups. The PEF values showed a nonsignificant difference between the groups (Table 2).

**Table 1:** Comparison of pre- and post-treatment values of FVC, FEV1, FVC/FEV1, and PEF within Group 1 (VRBT) and Group 2 (IST) FVC: forced vital capacity; FEV1: forced expiratory volume in one second; PEF: peak expiratory flow; VRBT: virtual reality breathing training; IST: incentive spirometry

Variables	Groups	Pretest		Posttest	
		Mean ± SD	Significance	Mean ± SD	Signicifance
FVC (L)	Group-1	1.28 ± 0.33	.001	1.93 ± 0.34	.001
	Group-2	1.45 ± 0.31	.001	1.77 ± 0.34	.001
FEV1 (L)	Group-1	1.39 ± 0.26	.001	1.97 ± 0.34	.001
	Group-2	1.48 ± 0.25	.001	1.83 ± 0.23	.001
FVC/FEV1 (%)	Group-1	64.35 ± 23.5	.001	82.07 ± 12.5	.001
	Group-2	62.28 ± 22.5	.001	74.50 ± 16.4	.001
PEF (L)	Group-1	2.23 ± 0.27	.001	2.73 ± 0.25	.001
	Group-2	2.28 ± 0.27	.001	2.72 ± 0.21	.001

**Table 2:** Comparison of Pre- and Post-treatment values of FVC, FEV1, FVC/FEV1, and PEF between Group 1 (VRBT) and Group 2 (IST) FVC: forced vital capacity; FEV1: forced expiratory volume in one second; PEF: peak expiratory flow; VRBT: virtual reality breathing training; IST: incentive spirometry

Variables	Groups	Number	Mean ± SD	F value	Significance
FVC (L)	1	16	0.644 ± 0.18	12.352	.001
	2	16	0.325 ±0.7		.001
FEV1 (L)	1	16	0.581 ± 0.19	1.002	.001
	2	16	0.350 ± 0.13		.001
FVC/FEV1 (%)	1	16	17.72 ± 6.6	5.50	.001
	2	16	12.22 ± 6.9		0.08
PEF (L)	1	16	0.500 ± 0.17	1.535	0.26
	2	16	0.444 ± 0.10		0.27

## Discussion

In this study, 32 children with spastic CP were treated with VRBT (Group 1; n = 16), IST (Group 2; n = 16). This study was done to determine which therapy was better for improving pulmonary function in children with spastic diplegic CP. FVC, FEV1, FVC/FEV1, and PEF were used to assess the pulmonary function in both groups.

The pre- and post-test results showed significant improvement in pulmonary function in both groups, indicating that both interventions effectively improved pulmonary function in the sample studied. When comparing the values of FVC, FEV1, FVC/FEV1, and PEF, a significant difference was found in the VRBT group. There was a significant improvement in the FVC and FEV1 values in the VRBT group, compared to the IST group, and there was more of an improvement in pulmonary function values in the VRBT group than the IST group. Earlier studies support our study results [15,16].

In the study by Joo et al., all the pulmonary parameters improved in the game-based breathing group compared to the control group, except for the FVC/FEV1, which showed little improvement [7]. The significant improvement was short-term for stroke patients in the game-based exercise group. The maximal inhalation and expiration performance was enhanced by repetitive and regular feedback from the respiratory program. The study showed that VRBT improved pulmonary function for stroke patients. There is an obvious side effect of changing the space of control of the children, which changes the learning process through biofeedback therapy, as per Rosenbaum et al. [17].

Additional benefits have been provided to non-cystic bronchiectasis through video game-based training, which improves muscle strength and endurance, as shown by Ungun et al. [8]. In their study, video game-based training also increased respiratory muscle strength. In addition, both aerobic and breathing video game-based training were effective in improving balance, but neither was superior.

A study by Lee et al. showed that respiratory training improved with the use of the feedback method, which involves continuous and repetitive inspiration and expiration [18]. Their study also showed a significant improvement in the pulmonary function parameters by respiratory training, except in the maximal expiratory pressure with very less samples. Therefore, no statistically significant improvement was found in the respiratory training through feedback. In the current study, it is clear that VR significantly improved pulmonary function for children with spastic diplegic CP. A general agreement has been reached among researchers that the treatment is less effective when carried out for less than six weeks [19]. 

The first limitation of our study was that the treatment effect was seen in the short term; future studies should focus on long-term effects. The next limitation was that only a few pulmonary function parameters like FVC, FVC/FEV1, and PEF were seen due to the availability of basic standard equipment; further studies can correlate other PFT parameters with advanced equipment. Another limitation is that we focused only on children with spastic diplegic CP; additional studies can be done for the other types of CP with gross motor function levels (IV and V). The final limitation is that variables like gender were not considered, which can be considered in future studies as the pulmonary function parameters vary between male and female children. Assessment of quality of life for children with CP also can be included in future studies.

## Conclusions

In this study, VRBT resulted in significant improvement in the FVC, FEV1, and FVC/FEV1 compared to the IST group. By including VRBT in pulmonary treatment, significant improvements can be achieved for children with spastic diplegic CP. It has been concluded that VR is a feasible and effective therapeutic method for improving pulmonary function in children with spastic diplegic CP. Providing the orientation about the instrument was difficult, but motivating the children enhanced the pulmonary functions. It forms an alternative treatment technique for children with CP as it is a quantifiable approach that improves the strength of the treatment.
